# Microglial pyroptosis as a therapeutic target after traumatic spinal cord injury: current progress and future directions

**DOI:** 10.3389/fimmu.2025.1649790

**Published:** 2025-08-22

**Authors:** Lei Shi, Qiheng Qian, Jiding Xie, Taoshuo Yang, Xinyu Zhao, Xiangqi Meng, Jingang Dai, Qiguan Jin

**Affiliations:** ^1^ College of Physical Education, Yangzhou University, Yangzhou, Jiangsu, China; ^2^ Suzhou Hospital of Traditional Chinese Medicine, Suzhou, Jiangsu, China; ^3^ Experimental Research Center, China Academy of Chinese Medical Sciences, Beijing, China; ^4^ College of Medical, Veterinary and Life Sciences, University of Glasgow, Glasgow, United Kingdom; ^5^ Xuzhou Hospital of Traditional Chinese Medicine, Xuzhou, Jiangsu, China

**Keywords:** traumatic spinal cord injury, pyroptosis, microglia, inflammation, NLRP3, GSDMD

## Abstract

Traumatic spinal cord injury (TSCI) is a devastating neurological condition with limited therapeutic options and a high likelihood of permanent disability. Among the multifaceted secondary injury mechanisms triggered by TSCI, pyroptosis—an inflammatory form of programmed cell death—has emerged as a key pathological process. In particular, microglial pyroptosis plays a pivotal role in exacerbating neuroinflammation and disrupting tissue homeostasis, thereby amplifying the secondary injury cascade. This review provides a comprehensive overview of the molecular pathways mediating microglial pyroptosis, including canonical (NLRP3–caspase-1–GSDMD), non-canonical (caspase-11–GSDMD), and atypical (caspase-3/8–GSDME/GSDMC) signaling. We also examine recent therapeutic strategies aimed at suppressing microglial pyroptosis—such as extracellular vesicle-based delivery systems, small-molecule compounds, and gene-targeted approaches—and assess their potential to enhance neurological and motor recovery following SCI. By elucidating both the pathological significance and therapeutic promise of microglial pyroptosis, this review offers novel perspectives on its translational potential as a target for spinal cord injury intervention.

## 1 Introduction

Traumatic spinal cord injury (TSCI) refers to denotes an abrupt, often irreversible disruption of spinal parenchyma precipitated by high-energy mechanical forces—such as falls, motor-vehicle collisions and sports trauma—which instantaneously destroy neurons, glia and the microvasculature, producing profound sensorimotor deficits and imposing substantial socioeconomic burdens (1, 2).

Pathologically, TSCI progresses through two distinct phases: primary injury and secondary injury (3). The primary injury arises directly from mechanical forces (e.g., compression, traction, laceration) that cause irreversible structural damage to spinal cord tissue (4). In contrast, secondary injury initiates rapidly after the primary insult and involves a complex, sustained cascade of pathophysiological events, including neuroinflammation, oxidative stress, excitotoxicity, apoptosis, pyroptosis, edema, and disruption of the blood-spinal cord barrier (BSCB). The progression of secondary injury can last from hours to weeks or longer, typically divided into acute (hours to 3 days), subacute (3 days to 2 weeks), and chronic (weeks to months) phases (5). Each phase may exacerbate the initial damage and impair long-term functional recovery. Notably, compared to the irreversible nature of primary structural damage, secondary injury exhibits greater plasticity and therapeutic potential, and timely interventions targeting secondary injury may reduce long-term neurological deficits.

Pyroptosis is a pro-inflammatory form of programmed cell death distinct from classical apoptosis (6). Its hallmark molecular features include the assembly of inflammasomes and activation of caspase-1, which cleaves substrates such as Gasdermin D (GSDMD) (7). The N-terminal fragment of GSDMD (GSDMD-NT) forms pores in the cell membrane, leading to rapid cell lysis and the release of cellular contents (8). Pyroptosis triggers the maturation and secretion of inflammatory mediators (e.g., IL-1β, IL-18), which induce intense local inflammation and exacerbate damage to adjacent cells.

Microglia, the resident immune cells of the central nervous system (CNS), play critical roles in immune surveillance, debris clearance, and synaptic pruning (9). Studies show that microglia are among the first cells to respond following spinal cord injury, undergoing morphological changes, migration, and phenotypic transformation to participate in inflammatory reactions at the injury site (10). Activated microglia exhibit a “double-edged sword” effect: they can promote repair by clearing necrotic debris and releasing neurotrophic factors, but may also aggravate the local inflammatory milieu through the secretion of pro-inflammatory cytokines such as TNF-α and IL-1β (11).

In the context of spinal cord injury, growing evidence highlights microglial pyroptosis as a pivotal event in secondary injury (12). As key contributors to post-injury inflammation, elevated pyroptosis in microglia is thought to worsen the neuroinflammatory environment (13). Numerous studies demonstrate significant upregulation of pyroptosis-related molecules (e.g., NLRP3 inflammasome components, cleaved GSDMD) in microglia after TSCI. Pyroptotic microglial death may also impair their beneficial roles in debris clearance and regenerative support. Thus, microglial pyroptosis is recognized as a critical link in the secondary injury cascade, profoundly impacting motor and neurological functional recovery.

## 2 Spinal cord injury and microglia

### 2.1 Spinal cord injury and secondary injury

During the acute phase of SCI, spinal cord ischemia, vasogenic edema and glutamate-mediated excitotoxicity inflict the primary insult, whereas neuroinflammation, mitochondrial dysfunction, overactive nitric-oxide-synthase (NOS), excessive apoptosis/necrosis, axonal degeneration and glial-scar formation synergistically hinder axonal remyelination and remodeling, ultimately dictating neurological prognosis (14). Minutes after trauma, an explosive inflammatory cascade releases damage-associated molecular patterns (DAMPs) that swiftly recruit and activate resident glia and peripheral immune cells within the CNS (15). Pro-inflammatory cytokines—IL-1β, IL-6 and TNF-α—rise steeply in tissue and cerebrospinal fluid within hours. Activated microglia and infiltrating macrophages are detectable in the parenchyma as early as 1 h, peak at 5–10 days and can persist for months (16). The diverse mediators released by these inflammatory cells collectively shape the secondary injury microenvironment, exacerbating pathological processes such as ischemia, edema, oxidative free radical accumulation, apoptosis, and pyroptosis (17). Timely curtailment of this cascade is therefore paramount for salvaging residual neural tissue and preserving function.

### 2.2 Activation of microglia and their associated roles in spinal cord injury

Microglia—the brain’s resident “sentinels” and “scavengers”—continually survey the parenchyma under homeostatic conditions (18). After SCI they are rapidly activated, becoming one of the earliest cellular responders (19). Within minutes-to-hours they enlarge, proliferate and migrate towards the lesion core. Activated microglia appear as early as 1 h, peak at 5–10 days and remain for weeks-to-months (20).

The activation state of microglia exhibits a dual nature: On one hand, excessive activation of microglia leads to the release of large amounts of pro-inflammatory mediators, exacerbating tissue damage (21). On the other hand, moderate activation facilitates debris clearance and secretion of neurotrophic factors, promoting tissue repair. Based on their activation states and functions, microglia are typically categorized into two phenotypes: the classically activated M1 phenotype and the alternatively activated M2 phenotype (22).

#### 2.2.1 Activation states of microglia

M1 microglia predominate during the acute phase of SCI and exhibit pro-inflammatory and neurotoxic effects (23). They secrete high levels of inflammatory mediators, such as IL-6, IL-12, and IFN-γ (24, 25), which trigger inflammatory cascades in neighboring cells, leading to severe neuronal and glial cell death and demyelination. M1 microglia also generate excessive reactive oxygen species (ROS) and proteases, causing further tissue damage (26).

In contrast, M2 microglia exert anti-inflammatory and neuroprotective roles by releasing anti-inflammatory cytokines (e.g., IL-10, IL-4, TGF-β) and growth factors (27–29). These mediators suppress inflammation and promote tissue repair and axonal regeneration. However, recent studies emphasize that the M1/M2 classification represents a spectrum rather than a strict dichotomy (30). However, recent studies suggest that microglial classification represents a continuum rather than two extreme, polarized phenotypes. With the advancement of technologies such as single-cell sequencing and spatial transcriptomics, microglia are now classified into multiple functional subtypes based on molecular characteristics, each with distinct nomenclature (31).

Homeostatic microglia refer to the resident microglia that maintain CNS homeostasis under physiological conditions—traditionally described as the “resting” state. Their marker genes include P2RY12, TMEM119, CX3CR1, SIGLEC-H, and HEXB (30).

Interferon-responsive microglia exhibit gene signatures induced by type I interferon stimulation, typically observed in acute inflammation or viral infection. However, studies have shown that this phenotype also exists in healthy mice, with notable sex-specific differences—male mice predominantly exhibit the interferon-responsive profile (high expression of the male-specific gene Eif2s3y), whereas females retain a homeostatic phenotype (high expression of the female X-linked gene Xist) (32).

Disease-associated microglia (DAM) were first identified in neurodegenerative conditions, characterized by the upregulation of genes involved in phagocytosis and lipid metabolism, such as APOE, TREM2, CD11c/ITGAX, and CLEC7A, accompanied by downregulation of homeostatic genes. This subtype is associated with lipid dysregulation and impaired clearance function and is mainly observed in neurodegeneration, demyelinating diseases, and late-stage acute injuries.

Proliferative-region-associated microglia (PAMG) are detected in neurogenic niches during development and participate in clearing apoptotic cells and promoting neurogenesis. Wang et al. found that PAMGs appear predominantly in the early acute phase of SCI (within ~3 days), characterized by genes involved in cell proliferation and stress response. These cells can be further divided into two subclusters: PAMG1, which highly expresses cell cycle regulatory genes (e.g., Mcm3, Cdk1) to promote proliferation; and PAMG2, which upregulates genes related to oxidative stress and inflammation (e.g., Tlr2, Cd5l, Ifi204), suggesting a potential role in counteracting injury-induced oxidative environments.

Meanwhile, injury-associated microglia (IaMG) are prominently enriched during the subacute phase post-injury. These are mainly divided into IaMG1 and IaMG2, both expressing inflammation-related genes such as Stat1, Cst7, and Cybb. Notably, the IaMG2 subset also upregulates genes associated with angiogenesis and axon regeneration (e.g., Nrp2, Fn1, Cxcr4, Rab7b), indicating its potential role in tissue repair and axonal regrowth. This highlights that post-injury microglial subtypes are not functionally homogeneous (30).

In addition, other subtypes have been proposed based on disease models, such as glioma-associated microglia (GAM), post-stroke microglia, and Parkinson’s disease-associated microglia. Some studies have noted overlapping features and lineage connections among different subtypes (33), and evidence suggests that microglia in varying activation states can migrate between regions as disease progresses (33, 34). These findings complicate nomenclature and experimental interpretation but underscore the remarkable plasticity of microglia and their ability to transition across diverse states depending on temporal and microenvironmental cues. Understanding these subpopulations is essential for elucidating the mechanisms by which microglia contribute to injury repair.

#### 2.2.2 Microglial efferocytosis after spinal cord injury

Microglial engulfment of dying cells and myelin debris is indispensable for establishing a pro-regenerative milieu in the CNS. This engulfment—termed efferocytosis—progresses through three coordinated steps: “find-me,” “eat-me,” and “digest” signals that sequentially attract, engage, and remove dying cells (35). Briefly, apoptotic cells emit chemo-attractants that bind dedicated receptors on phagocytes, triggering engulfment; the resulting phagosome then fuses with lysosomes, where the cargo is enzymatically degraded (36).

Efferocytosis therefore represents a pivotal checkpoint in inflammation resolution. By-products generated during digestion actively re-programme immune cells, steering them toward pro-resolving phenotypes and restoring tissue homeostasis (35). After brain injury, efferocytosis in the CNS is often suppressed. Notably, EphA4 overexpression in microglia inhibits the P-ERK/P-Stat6/MERTK signaling axis (37). By contrast, microglia enriched for MERTK display heightened efferocytosis, foster oligodendrocyte regeneration, and improve functional outcome in demyelinating models (38). Likewise, Gas6 limits pro-inflammatory microglial activation and curtails microglia–astrocyte crosstalk, thereby attenuating post-SCI inflammation and glial-scar formation (39).

Multiple studies have shown that enhancing microglial phagocytic capacity improves outcomes in ischemic stroke, subarachnoid hemorrhage, and related conditions, likely through mechanisms involving the reduction of neuronal injury and modulation of CNS inflammation (40, 41). Some researchers have proposed that microglial phagocytic capacity is closely tied to their activation state. During efferocytosis, microglia may also adopt a pro-resolving phenotype, secreting anti-inflammatory cytokines such as TGF-β and IL-10 to suppress secondary inflammation and maintain tissue homeostasis (42, 43). Additionally, some studies have employed a strategy combining neutrophil membrane-derived vesicles and a “Trojan Horse” system to promote nerve regeneration and modulate inflammation after SCI through efferocytosis. This effect is mediated by the reprogramming of immune cells and regulation of the immune cascade (44). Collectively, these data underscore efferocytosis as a central driver of immune resolution and tissue repair in SCI. Therapeutic reinforcement of microglial efferocytosis thus offers a compelling avenue for improving neurological outcome.

## 3 Pyroptosis pathways following spinal cord injury

### 3.1 Classical caspase-1-dependent pathway

In the canonical pathway, pyroptosis is initiated by multi-protein inflammasomes—most notably NLRP3—that sense danger-associated molecular patterns (DAMPs) liberated after primary mechanical trauma (45). A prototypical signal is extracellular ATP, which binds microglial P2X7 receptors, drives K^+^ efflux, and thereby activates the NLRP3 inflammasome in SCI (46). High mobility group box 1 (HMGB1), a nuclear protein under physiological conditions, is upregulated in damaged neurons and microglia following SCI and can bind to receptors such as TLR2/4, thereby promoting M1-type polarization of microglia and increasing the release of pro-inflammatory mediators (47). Cellular stress increases mitochondrial permeability; oxidized mtDNA escapes into the cytosol and directly couples to NLRP3, driving inflammasome assembly (48). In addition, SCI-induced cell damage can release other DAMPs such as heat shock proteins (e.g., HSP70, HSP90), S100 proteins, and related molecules. These too are recognized by pattern recognition receptors and contribute to sterile inflammation (49, 50)^-^ (51). Collectively, ATP, HMGB1, and mtDNA represent well-characterized DAMPs in the context of SCI, corresponding to the release of metabolic, nuclear, and genetic materials, respectively. These molecules engage distinct receptors and pathways to drive NLRP3 inflammasome-mediated neuroinflammation.

NLRP3 inflammasome activation proceeds in two steps: the priming/transcriptional signal and the activating signal. The priming/transcriptional signal is initiated by DAMPs or other stimuli that activate transcriptional pathways such as NF-κB, resulting in upregulated transcription and translation of NLRP3 and its downstream pro-inflammatory cytokine precursors, including pro-IL-1β and pro-IL-18 (52). This step elevates the cellular abundance of inflammasome components and sensitizes the NLRP3 complex to activation, involving adapter proteins such as Myeloid differentiation primary response 88 (MyD88), Interleukin-1 receptor-associated kinase 1 (IRAK-1), TIR-domain-containing adaptor-inducing interferon-β (TRIF), and Fas-associated protein with death domain (FADD) (53, 54). The activating signal is closely tied to the aforementioned DAMPs and directly induces NLRP3 inflammasome assembly and activation of effector molecules such as caspase-1 (55). This second signal is often associated with ion fluxes, particularly potassium efflux and calcium influx, which are considered potential upstream events in NLRP3 activation (53, 56). In acute-to-subacute SCI, NLRP3–caspase-1 signaling surges in microglia and constitutes a linchpin of secondary degeneration (57). Excessive pyroptosis depletes protective microglia and floods the parenchyma with pro-inflammatory mediators, jeopardizing neuronal survival. Pharmacological or genetic inhibition of NLRP3 therefore constitutes a promising strategy to blunt neuroinflammation and foster recovery (58).

### 3.2 Non-canonical pyroptosis pathway mediated by Caspase-4/5/11

In the non-canonical route, human caspase-4/-5 (murine caspase-11) are directly engaged by cytosolic lipopolysaccharide (LPS), bypassing canonical inflammasome sensors (59). LPS docking to their CARD domains triggers rapid oligomerization and auto-activation of these caspases. The activated caspases cleave the linker region of Gasdermin D (GSDMD), releasing the N-terminal fragment (GSDMD-NT). This fragment inserts into the cell membrane, forming pores that trigger pyroptotic cell lysis (60).

While Caspase-4/5/11 do not directly process pro-IL-1β or pro-IL-18, the membrane pores formed by GSDMD-NT cause potassium ion efflux and other cellular disturbances (60, 61). These changes indirectly activate the NLRP3 inflammasome, leading to Caspase-1-dependent maturation and release of IL-1β and IL-18. Consequently, the non-canonical pathway often synergizes with the classical pathway, amplifying the inflammatory cascade (62). Caspase-11 can also cleave the large-pore channel pannexin-1, leading to massive ATP release from the cell. The extracellular ATP then activates the P2X7 receptor, which further triggers potassium efflux, thereby promoting the activation of the NLRP3 inflammasome (63). Mice lacking P2X7 or pannexin-1 exhibit greater resistance to LPS, indicating that this signaling axis is essential for caspase-11-dependent non-canonical pyroptosis. Such cross-talk intensifies neuroinflammation after SCI, underscoring the intricate tapestry of pyroptotic signaling in secondary pathology.

### 3.3 Atypical pyroptosis mediated by caspase-3/8

Mounting evidence indicates that the executioner caspases-3 and caspases-8, historically viewed as apoptotic proteases, can instigate pyroptosis via unconventional cleavage of specific gasdermins, thereby constituting inflammasome-independent “atypical” pathways (64).

Caspase-3 is recognized as the protease executing apoptosis (65). However, in cells with high GSDME expression, caspase-3 can cleave GSDME, releasing its N-terminal pore-forming domain. This shifts apoptosis toward pyroptosis-like lytic cell death (66). Research indicates that GSDME acts as a “molecular switch,” triggering membrane pore formation and inflammatory mediator release in caspase-3-activated cells (67). Post-SCI, elevated GSDME levels are observed, and its suppression reverses neuroinflammatory exacerbation (68). Microglia express GSDME under pathological conditions, undergoing caspase-3-dependent GSDME cleavage and pyroptotic death upon injury (69).

Caspase-8, a key enzyme in the extrinsic apoptosis pathway, has recently been shown to cleave Gasdermin C (GSDMC) under specific inflammatory conditions (e.g., high TNF-α and IFN-γ levels), inducing pyroptosis in cancer cells (70). Metabolite α-ketoglutarate (α-KG)-induced pyroptosis via death receptor DR6 and caspase-8-mediated GSDMC cleavage has also been reported, dependent on ROS elevation and acidic microenvironments (71). Additionally, caspase-8 can inefficiently cleave GSDMD or promote inflammasome activation, further linking it to pyroptosis (72). Muendlein et al (73) recently proposed the concept of “efferoptosis,” referring to a form of macrophage death termed “macrophage efferoptosis” induced by TNF during efferocytosis. In this process, TNF-activated macrophages undergo TRIF/caspase-8/GSDMD-dependent cell death after engulfing neutrophils. Notably, IL-1β maturation in this context does not rely on the NLRP3 inflammasome but instead occurs via direct cleavage by caspase-8. This suggests that a similar pathway may also be involved in microglial pyroptosis following SCI.

### 3.4 ROS-mediated pyroptosis

Excess reactive-oxygen species (ROS) generated after SCI constitute a pivotal trigger of inflammasome activation and ensuing pyroptosis. ROS potentiate NLRP3 oligomerization by inducing thiol oxidation, ionic flux and mitochondrial dysfunction (74, 75). Studies show that pathological events post-SCI, such as hemorrhage, hypoxia, and iron ion release, amplify ROS production (76). Excessive ROS triggers NLRP3 inflammasome-mediated pyroptosis. Additionally, ROS indirectly activate the inflammasome by disrupting lysosomal membranes (causing lysosomal enzyme leakage) and damaging mitochondria (releasing mitochondrial DNA and other DAMPs) (77). In microglia, uncontrolled ROS levels persistently stimulate caspase-1/GSDMD-dependent pyroptosis, releasing inflammatory mediators that exacerbate neurological damage.

Targeting oxidative stress via antioxidant therapies has emerged as a promising strategy to suppress pyroptosis and mitigate inflammation (78). For instance, Cynarin inhibits microglial pyroptosis in SCI models by enhancing Nrf2 antioxidant signaling, reducing ROS levels, and suppressing NLRP3 inflammasome assembly (79). This mechanism highlights the therapeutic potential of antioxidants in modulating pyroptosis and improving outcomes in SCI.

Mitochondrial Damage and Pyroptosis

### 3.5 Mitochondrial damage-mediated pyroptosis

**Figure 1:**
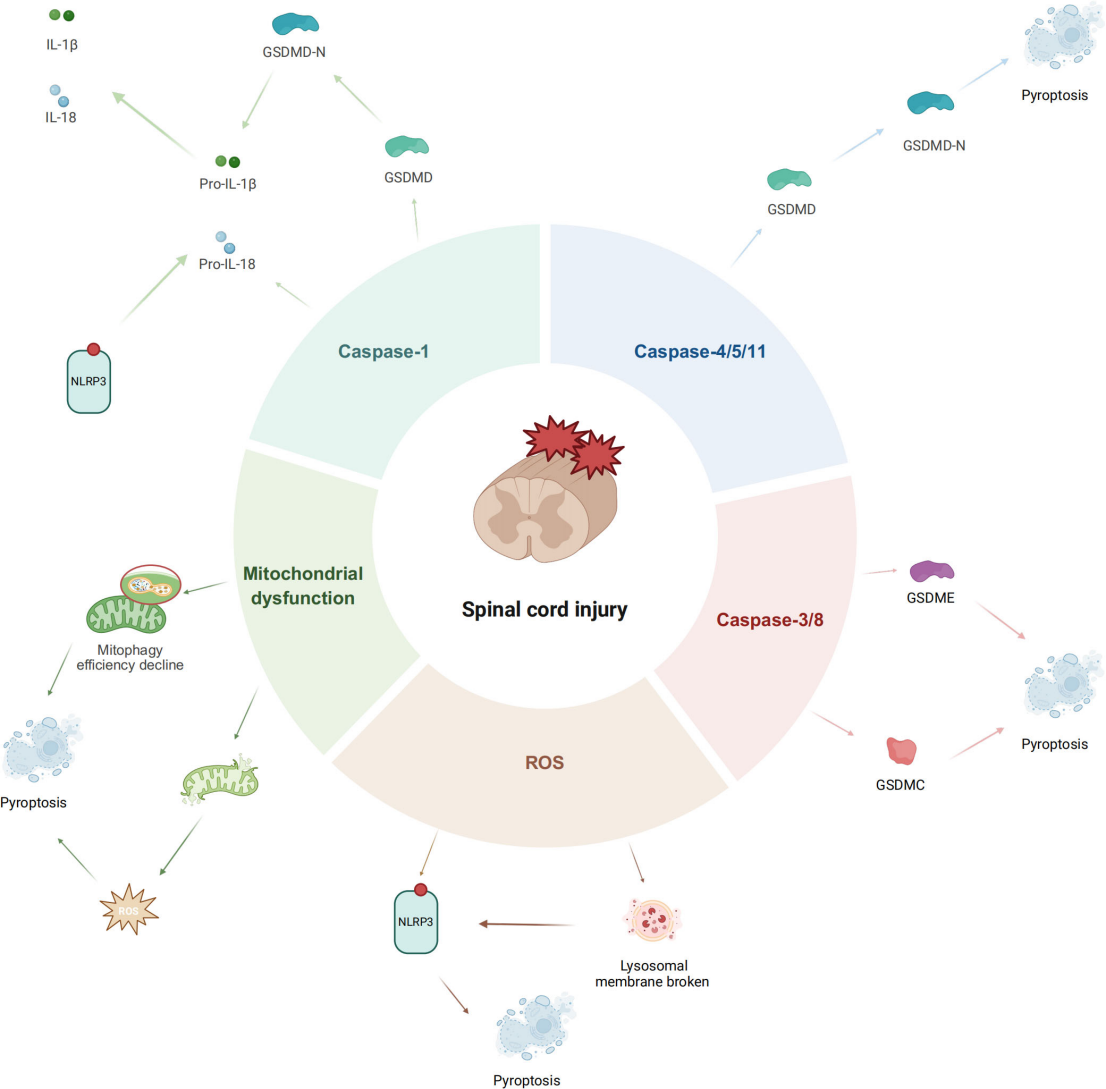
Diagram of pyroptosis pathway following spinal cord injury (created by Biorender).

As metabolic powerhouses, mitochondria are intimately linked to cell-death pathways; injury-induced dysfunction prompts excess ROS production and releases mtDNA, oxidized cardiolipin, and other DAMPs into the cytosol (80–82). These molecules act as DAMPs to activate inflammasomes such as NLRP3 or AIM2, triggering caspase-1-mediated pyroptosis (83, 84). Targeting this mechanism, enhancing mitophagy (selective autophagy of mitochondria) to clear damaged mitochondria has emerged as an effective strategy to suppress pyroptosis. For instance, the natural compound Betulinic acid promotes autophagy and mitophagy, clearing dysfunctional mitochondria and reducing ROS levels, thereby significantly inhibiting microglial pyroptosis during SCI (85). Similarly, Urolithin A alleviates microglial pyroptosis and inflammation by enhancing mitophagy in injured tissues (86). These findings underscore the importance of maintaining mitochondrial homeostasis to inhibit pyroptosis and mitigate secondary injury in SCI (**Figure 1**).

## 4 Targeted modulation of microglial pyroptosis to promote neurological and motor recovery post-SCI

Currently, there are no clinically approved pyroptosis-targeted interventions for SCI. However, preclinical studies have demonstrated the critical importance of targeting microglial pyroptosis to improve neurological and motor functional recovery following SCI. Strategies such as cell transplantation, extracellular vesicles derived from other cell sources, synthetic drugs, natural compounds, and genetic modulation of key pyroptosis regulators have shown significant therapeutic potential (Shown **Table 1** for details).

**Table 1:** Summary table of studies on targeted interventions in post-SCI microglial pyroptosis and their impact on prognosis.

Treatments	Key factor	Experimental models	Methods of administration	Mechanisms of action	Therapeutic effects	Reference
Apocynin	Advanced oxidation protein products(AOPPs)	C5 hemi-contusion	Intraperitoneal injection	Nox4-ROS-NLRP3-GSDMD	The NADPH oxidase inhibitor apocynin suppresses AOPP-induced microglial pyroptosis via the ROS-dependent MAPK-NF-κB signaling pathway and NLRP3-GSDMD pathway following spinal cord injury (SCI), thereby improving SCI prognosis.	(101)
Exosomes	miR-709	T8 spinal cord contusion	Intrathecal injection	NKAP	Treg cells suppress microglia pyroptosis by secreting the exosome miR-709, which inhibits NKAP expression. Injection of Treg cells or Treg cell-derived exosomes inhibited microglia pyroptosis activation, resulting in improved functional recovery after spinal cord injury.	(92)
SC79	CD73(NT5E)	Spinal crush injury at the T8‐T9	Intraperitoneal injection	PI3K/AKT/Foxo1	CD73 alleviates GSDMD‐mediated pyroptosis through inhibiting PI3K/AKT/Foxo1 signaling. CD73 promotes an increase in the concentration of extracellular adenosine after injury, increases PI3K/AKT activation through the A2B adenosine receptor, thereby blunting NLRP3 inflammasome activation and reducing GSDMD transcription. The accumulation of HIF‐1α after spinal cord injury facilitates the upregulation of CD73, while the overexpressed CD73 promotes the further aggregation of HIF‐1α through adenosine‐A2BAR‐p38 cascade, forming a positive feedback regulation.	(111)
Lycium barbarum glycopeptide	modulating docosahexaenoic acid	Left-sided spinal cord transection at the T12 vertebral level	Orally(nasogastric tube)	MAPK-NF-κB	LbGp stimulates microglia to produce DHA by regulating the key enzymes FADS1 and FADS2 in microglia, and thus, DHA can improve neuro inflammation by inhibiting the MAPK/NF-kB and pyroptosis pathways group	(102)
pLVX‐dead‐box helicase 3 X‐linked	TLR4	Spinal crush injury at the T8‐T9	Intrathecal injection	JAK2/STAT1/DDX3X/NLRP3	TLR4 promotes microglial pyroptosis by activating the STAT1/DDX3X/NLRP3 signalling axis after SCI in vivo and in vitro. BGN is an important molecule that mediates the pro‐pyroptotic role of TLR4.	(112)
HSPA1A-overexpressing lentivirus	Heat shock protein family A member 1A (HSPA1A) DUSP1	T8 spinal cord contusion	Intrathecal injection	MAPK	HSPA1A was exerted through upregulation of DUSP1 to further inhibit the MAPK pathway	(113)
Exosomes	miR-21a-5p	T8 spinal cord contusion	Intrathecal injection	miR-21a-5p/PELI1	BMSC-derived exosomes enhanced autophagy and suppression of pyroptosis in macrophage/microglia, mediated by the miR-21a-5p/PELI1 axis	(93)
Lupenone	NLRP3	T8 spinal cord contusion	Intraperitoneal injection	NF-κB	Lupenone improves the local inflammatory microenvironment by inhibiting neuroinflammation via suppression of the NF-κB signaling pathway. The results indicated that Lup alleviates neuroinflammation by modulating activation of inflammasome and subsequent microglial polarization and pyroptosis.	(103)
Pyrrolidine dithiocarbamate(bmal1 knockout )	MMP9	Spinal crush injury at the T8‐T9(bmal1 KO)	Slowly microinjected to a depth of ~1 mm at the site of spinal cord injury	NF-κB /MMP9	Bmal1 regulates the NF-κB /MMP9 pathway to reduce microglial pyroptosis and thereby reduce secondary spinal cord injury	(114)
Exosomes	let-7b-5p	T8~T9 spinal cord contusion	Intrathecal injection	LRIG3	IPSC-NSCs and their exosomes effectively suppress pyroptosis and neuroinflammation in microglial/macrophages subjected to SCI and LPS stimulation. These interventions alleviate the formation of glial scars, maintain the integrity of myelin, and facilitate the growth of axons, ultimately restoring functional abilities in SCI mice.	(94)
shRNA-FANCC adeno-associated virus	Fanconi Anemia Group C complementation group gene	T10 spinal cord contusion	Intrathecal injection	p38/NLRP3	Increased expression of FANCC in SCI mice and LPS-stimulated microglia markedly inhibited pyroptosis and neuroinflammation via blocking the p38/NLRP3 pathway	(115)
Resveratrol	miR-124-3p/DAPK1(Death‐associated protein kinase 1)	Spinal Cord Ischemia-Reperfusion Injury	Intraperitoneal injection	NLRP3/Caspase-1/GSDMD	Resveratrol elevates miR-124-3p levels targeting DAPK1, modulates the NLRP3/Caspase-1/GSDMD pathway, suppresses pyroptosis, and mitigates SCI.	(104)
lncRNA-F630028O10Rik	TLR4	Spinal crush injury at the T8‐T9	Intrathecal injection	PI3K/AKT	TLR4 was activated following SCI and promoted the expression of lncRNA-F630028O10Rik. This lncRNA functioned as a ceRNA for miR-1231-5p/Col1a1 axis and enhanced microglial pyroptosis after SCI by activating the PI3K/AKT pathway.	(95)
CerS5 siRNA	Ceramide synthase 5	T10 spinal cord contusion	Intrathecal injection	CerS5/NLRP3	Inhibiting microglial CerS5 expression after SCI effectively mitigates neuroinflammation by suppressing microglial pyroptosis, thereby exerting neuroprotective effects. This process involves C16 ceramide, a downstream product of CerS5 metabolic pathway, which activates the NLRP3 signaling pathway in a manner dependent on Pla2g7 and NFκB.	(116)
DPSC-CM	Interleukin-1β	T10 spinal cord contusion	Intraperitoneal injection	NLRP3/caspase-1/IL-1β	Human dental pulp stem cells can reduce microglial pyroptosis by inhibiting the NLRP3/caspase-1/interleukin-1β pathway, thereby promoting the recovery of neurological function after spinal cord injury.	(96)
circ0000381-siRNA	miR-423-3p	T10 spinal cord contusion	-	circ0000381/miR-423-3p/NLRP3	Following an earlier increase of NLRP3 and GSDMD, circ0000381 upregulation may be a compensatory change to limit microglial/macrophage pyroptosis after SCI. Moreover, circ0000381 can bind to miR-423-3p and act as an endogenous sponge to inhibit miR-423-3p activity, thus attenuating spinal microglial/macrophage pyroptosis.	(97)
Exomoses	miRNA-22	Spinal crush injury at the T10	intrathecal injection	miRNA-22/GSDMD	miRNA‐22 can inhibit the pyroptosis of microglia. The combination of MSCs‐EV and miRNA‐22 can further inhibit the neuroinflammatory response after SCI, thereby improving the neurological function after SCI in rats.	(98)
shRNA(*shCebpb*、*shFcgr1)*	CAAT/Enhancer Binding Protein β	T9-T10 spinal cord contusion	Intrathecal injection	C/EBPβ-Fcgr1	The C/EBPβ-Fcgr1 axis induces neuroinflammatory responses by activating microglia pyroptosis following spinal cord injury.	(117)
Celastrol	NLRP3	Spinal crush injury at the T10	Intraperitoneal injection	NFκB/p-p65	Celastrol can attenuate the inflammatory response of the spinal cord after SCI, which is associated with inhibition of microglial activation and pyroptosis pathway.	(105)
Cynarin	Nrf2	T9 cords by a spinal cord impactor	Intraperitoneal injection	Nrf2/ROS/NLRP3	Cynarin inhibited the assembly of NOD-like receptor thermal protein domain associated protein 3 (NLRP3) inflammasome by Nrf2-dependent expression to attenuate microglial pyroptosis and neuroinflammation.	(79)
exo-sh-RMRP	SIRT1	Spinal crush injury at the T10	Intravenous Injections	EIF4A3/SIRT1	OM-MSCs-Exo mitigated microglial pyroptosis and promoted motor function recovery after SCI by delivering lncRNA RMRP. Further analysis elucidated that the inhibitory roles of exosomal lncRNA RMRP on microglial pyroptosis are dependent on EIF4A3/SIRT1 signaling.	(99)
AAV-GPx3	GPx3	C5 contusion	Intraspinal administration at the site of spinal cord injury.	IRAK4/ROS/NLRP3	GPx3 plays a critical role in SCI by inhibiting microglial pyroptosis via the IRAK4/ROS/NLRP3 signaling pathway. GPX3 PROMOTES FUNCTIONAL RECOVERY AFTER SCI 13	(75)
Oxindole/imidazole compound (C16)	protein kinase R (STAT1)	T8-T9 spinal cord contusion	Intraperitoneal injection	PKR/STAT1/NLRP3	PKR inhibition suppressed ER stress and NLRP3 inflammasome-related pyroptosis in microglia partly through suppressing STAT1	(118)
Kaempferol	NLRP3	C5 hemi-contusion	Intraperitoneal injection	NLRP3	Kaempferol could inhibite the pyroptosis related proteins (NLRP3 Caspase-1 p10 ASC N-GSDMD) and reduce the release of IL-18 and IL-1β.	(106)
TRIM32 overexpressing lentivirus	NEK7	Spinal crush injury at the T9-T10	Intraspinal administration at the site of spinal cord injury.	NEK7 reversed the inhibition of pyroptosis induced by TRIM32 in a ubiquitylation-dependent manner	TRIM32 inhibits microglia pyroptosis by facilitating the ubiquitylation of NEK7 at the K64 site, thereby alleviating the progression of SCI. The findings suggest that TRIM32 has the potential to be a therapeutic target of SCI.	(119)
Kanglexin	Pka	T9 spinal cord contusion	Persral	PKA/NF-κB	Klx modulates microglial polarization and pyroptosis via the PKA/NF-κB signaling pathway.	(107)
Taxifolin	NLRP3	Spinal crush injury at the T9	Persral	PI3K/AKT	PI3K/AKT signaling pathway participates in microglial pyroptosis after SCI	(108)

### 4.1 Cell transplantation and extracellular vesicle-based interventions

In recent years, cell transplantation and extracellular vesicle (EV)-based drug delivery technologies have rapidly advanced in the field of regenerative medicine, emerging as third-generation “biological therapeutic” strategies following small-molecule drugs and genetic engineering (87). According to the International Society for Extracellular Vesicles (ISEV), EVs are lipid bilayer-enclosed particles (including exosomes and microvesicles) naturally released by cells, capable of carrying diverse bioactive cargo (88). In addition to miRNAs or circRNAs, EVs can deliver proteins, lipids, and other therapeutic factors that aid in spinal cord repair. Compared to traditional pharmaceuticals, these approaches can cross the blood-spinal cord barrier, achieve precise delivery to lesions, and remodel the damaged microenvironment through multi-target, network-based regulation, balancing high efficacy with controllability (89, 90). In animal studies and early clinical trials for neurological disorders, stem cells and their derived extracellular vesicle have demonstrated potential in promoting neuroprotection, inflammation modulation, axonal regeneration, and functional recovery (91). Notably, extracellular vesicle inherently offer advantages such as low immunogenicity, feasibility for large-scale production, and adaptability to engineering modifications, thereby enabling safe and repeatable administration.

Regulatory T cells (Tregs) suppress microglial pyroptosis by secreting exosomal miR-709, which down-regulates NKAP; administering either Tregs themselves or their extracellular vesicles blocks microglial pyroptotic activation and ultimately improves functional recovery after SCI (92). Extracellular vesicles derived from bone-marrow mesenchymal stem cells (BMSCs) deliver miR-21a-5p, which enhances PELI1-dependent autophagy and thereby inhibits microglial pyroptosis (93). Induced pluripotent stem-cell–derived neural stem cell (iPSC-NSC) extracellular vesicles can package and transfer let-7b-5p to modulate LRIG3 expression, reducing microglia/macrophage pyroptosis and boosting motor recovery in mice after SCI (94). lncRNA-F630028O10Rik, released in extracellular vesicles following TLR4 activation after SCI, heightens microglial pyroptosis through the PI3K/AKT pathway (95). Transplantation of human dental-pulp stem cells decreases microglial pyroptosis via the NLRP3/caspase-1/IL-1β axis, thereby promoting neurological recovery after SCI (96). While circ0000381 is up-regulated after SCI, miR-423-3p declines; silencing circ0000381 elevates miR-423-3p and increases microglia/macrophage pyroptosis (97). Mesenchymal-stem-cell extracellular vesicles loaded with miRNA-22 suppress microglial pyroptosis in rats following SCI (98). Exosomal lncRNA RMRP from olfactory-mucosa mesenchymal stem cells mitigates microglial pyroptosis and enhances motor recovery through the EIF4A3/SIRT1 pathway (99). In addition, miR-146a, up-regulated via Nrf2 after SCI, down-regulates GSDMD in microglia, thereby restraining their pyroptosis (100). Neutrophil membrane vesicles combined with a composite fiber scaffold reprogram microglial phenotype and metabolism during inflammation, regulating the innate immune cascade to reduce neuroinflammation and promote neural regeneration (44). This scaffold mimics an “efferocytosis-like” mechanism whereby the EVs are endocytosed by macrophages/microglia, reprogramming them towards a pro-regenerative phenotype and significantly promoting nerve fiber regeneration after SCI. his strategy exemplifies how combining biomaterial scaffolds with EV-mediated immune modulation can synergistically coordinate inflammatory resolution and tissue repair in SCI.

### 4.2 Pharmacological and small-molecule interventions

Small-molecule drugs and natural products are regarded as one of the most clinically translatable intervention strategies because their chemical structures are well-defined, their quality is controllable, and their routes of administration are flexible. In recent years, numerous bioactive constituents derived from medicinal herbs or diet have been shown to cross the blood–brain/spinal barriers, scavenge ROS, modulate the immune-inflammatory network and promote axonal regeneration—offering multiple-target advantages. Alongside technological advances, a series of newly synthesized small molecules have also exhibited excellent pharmacokinetic properties and selective microglial targeting, providing a rich pool of lead compounds for the precision treatment of nervous-system disorders.

The NADPH-oxidase inhibitor apocynin blocks AOPP-induced microglial pyroptosis after SCI via ROS-dependent MAPK–NF-κB and NLRP3–GSDMD pathways, thereby improving outcomes (101). Lycium barbarum glycopeptide (LbGp) up-regulates the key enzymes FADS1 and FADS2 in microglia to boost DHA production and, by suppressing the MAPK/NF-κB and pyroptosis cascades, mitigates neuro-inflammation and enhances recovery (102). Lupenone diminishes IκBα activation and p65 nuclear translocation; by modulating NF-κB it inhibits NLRP3-inflammasome activity, reduces microglial pyroptosis and alleviates motor deficits after SCI (103). Resveratrol elevates miR-124-3p, which targets DAPK1 and down-regulates the NLRP3/Caspase-1/GSDMD axis, thereby lowering microglial pyroptosis (104). Celastrol suppresses microglial pyroptosis after SCI through the NF-κB/p-p65 pathway (105). Cynarin attenuates microglial pyroptosis post-SCI by up-regulating Nrf2 (75). Lupeol activates mitophagy via the AMPK–mTOR–TFEB pathway and strengthens Na^+^/K^+^-ATPase activity, inhibiting microglial pyroptosis and slowing SCI progression. Kaempferol curbs ROS generation by inhibiting NADPH oxidase-4 and restrains microglial pyroptosis through the MAPK–NF-κB pathway (106). Kanglexin (Klx), an anthraquinone compound, enhances PKA phosphorylation while inhibiting NF-κB and IκBα phosphorylation, thus limiting NF-κB nuclear translocation and NLRP3-inflammasome-induced microglial pyroptosis (107). Taxifolin targets PI3K/Akt signaling, lessens neuro-inflammation, promotes axonal regeneration and lowers microglial pyroptosis, thereby improving functional outcomes after SCI (108).

### 4.3 Targeted gene intervention

With the rapid advances of gene-editing platforms such as CRISPR/Cas and TALEN, manipulating specific genes within the CNS has moved quickly from simple “proof-of-concept” studies to bona-fide functional interventions (106). Compared with conventional small-molecule or protein inhibitors, genome editing can silence or activate pathogenic/protective genes with high precision, efficiency and durability, providing a highly specific tool for modulating the inflammatory cascade and remodeling the micro-environment (109). When coupled with delivery vehicles that cross the blood–brain barrier—such as recombinant adeno-associated virus (rAAV) and lipid-nanoparticle (LNP) systems—gene editing has already shown longer-lasting efficacy and controllable safety profiles than pharmacological therapies in multiple models of neuro­degenerative disease and SCI (110). Consequently, targeted gene intervention has become a major developmental direction for regulating microglial pyroptosis, mitigating secondary SCI, and treating other CNS disorders.

CD73 (ecto-5′-nucleotidase/NT5E) – an AMP-hydrolyzing ectoenzyme that converts extracellular ATP to adenosine. CD73 knock-down attenuates GSDMD-mediated pyroptosis by suppressing PI3K/AKT/Foxo1 signaling. After SCI, HIF-1α accumulation up-regulates CD73; in turn, CD73 over-expression amplifies HIF-1α via an adenosine–A2B receptor–p38 cascade, forming a positive-feedback loop (111). TLR4 – drives microglial pyroptosis after SCI through the STAT1/DDX3X/NLRP3 axis. Both TLR4 knockout and supplementation with biglycan (BGN) reverse this effect (112). HSPA1A (Heat-shock protein A member 1A) – a molecular chaperone highly induced after TSCI. Over-expression via lentiviral vectors up-regulates DUSP1 and inhibits MAPK signaling, thereby reducing microglial pyroptosis (113). Bmal1 – a core circadian-clock gene. Bmal1 limits microglial pyroptosis and secondary SCI by down-regulating the NF-κB/MMP9 pathway (114). FANCC (Fanconi-anemia complementation group C) – previously considered anti-inflammatory; its targeted inhibition lowers microglial pyroptosis via the p38/NLRP3 pathway (115). CerS5 (Ceramide-synthase 5) – silencing CerS5 in microglia alleviates neuro­inflammation by suppressing pyroptosis. The mechanism involves the downstream product C16-ceramide, which activates the NLRP3 pathway through Pla2g7 and NF-κB (116). C/EBPβ (CCAAT/enhancer-binding protein β) – linked to inflammatory status in neuro­degeneration; its knock-down diminishes microglia-mediated neuro­inflammation by repressing Fcgr1 transcription (117). GPx3 (Glutathione-peroxidase 3) – an antioxidant enzyme. GPx3 silencing elevates ROS and increases IRAK4 and pro-inflammatory cytokines, thereby enhancing microglial pyroptosis (75). PKR (Protein-kinase R) – a type I ER-membrane kinase traditionally associated with ER stress. In SCI it modulates microglial pyroptosis via the STAT1 pathway (118). TRIM32 – an E3-ubiquitin ligase. TRIM32 inhibits microglial pyroptosis by promoting ubiquitination of NEK7 at lysine 64, slowing SCI progression (119).

## 5 Discussion and future directions

Compelling evidence now demonstrates that microglial pyroptosis orchestrates secondary degenerative cascades after SCI (13, 120). As resident immune sentinels of the central nervous system, microglia are rapidly recruited and activated within minutes of trauma, initiating a robust inflammatory response. During this lytic form of programmed cell death, microglia undergo rapid swelling and lysis, releasing inflammatory mediators (e.g., IL-1β, IL-18) and cellular contents. These mediators exacerbate local neuroinflammatory cascades, causing further damage to adjacent neurons and oligodendrocytes and amplifying secondary tissue damage (121). Conversely, multiple pre-clinical studies show that genetic or pharmacological suppression of microglial pyroptosis markedly attenuates neuroinflammation, limits cellular loss and accelerates locomotor recovery post-SCI (57, 75, 116).

Nano-sized extracellular vesicles have emerged as versatile carriers for anti-pyroptotic cargo. extracellular vesicles, with their small size and low immunogenicity, can penetrate the blood-spinal cord barrier and evade mononuclear phagocyte clearance (122, 123). Studies utilizing stem cell-derived exosomes as carriers for delivering anti-pyroptosis molecules have shown efficacy (124). The advantages of extracellular vesicles include targeted delivery and tissue permeability, but challenges remain in their high preparation/purification costs, complex processes, and lack of standardized quality control. Critically, batch-to-batch consistency in bioactivity and clarity of active components must be resolved before clinical translation.

A growing pharmacopeia of small-molecule inhibitors, antioxidant polyphenols and natural compounds can attenuate microglial pyroptosis *in vivo* (125, 126). Anti-inflammatory or antioxidant small molecules (e.g., Taxifolin, resveratrol, luteolin) have been shown to attenuate neuroinflammation and suppress microglial pyroptosis post-SCI. These drugs benefit from mature production processes and ease of administration, with some natural compounds exhibiting favorable biosafety (79, 105). Nevertheless, their pleiotropic targets and limited cell specificity raise concern regarding off-target immunosuppression, and systemic delivery must still overcome the blood–spinal cord barrier to achieve therapeutic concentrations while minimizing adverse effects.

Gene-based interventions, such as knockout or silencing of key nodes in pyroptosis pathways, provide robust evidence in animal studies (127, 128). Adeno-associated viruses (AAVs) or lipid nanoparticles delivering shRNA/siRNA have also emerged as tools to inhibit microglial pyroptosis (129, 130). Gene therapies offer high specificity and durable effects by targeting critical pyroptosis molecules. However, clinical translation faces hurdles, including immune responses to delivery vectors, safety/ethical concerns regarding gene editing, and ensuring cell-specific targeting without compromising systemic immunity (131, 132). Furthermore, these approaches are costly, technically demanding, and logistically challenging in acute injury scenarios.

Each intervention modality for modulating microglial pyroptosis carries distinct advantages and limitations. EV-based biological therapies (including cell transplants and EV carriers) enable targeted multi-factorial modulation of the injury microenvironment, with the ability to cross the BSCB and high biocompatibility; however, their production is costly and complex, and standardization of contents and potency remains challenging. Small-molecule drugs, by contrast, are easy to administer and can broadly suppress inflammation or oxidative stress; they benefit from well-established manufacturing and generally good safety profiles, but often lack cell-type specificity and must effectively penetrate into the spinal cord, raising concerns about off-target effects. Gene-editing and gene-silencing approaches (e.g. CRISPR/Cas9 or RNAi therapies) precisely target key pyroptosis-related genes with potentially long-lasting effects, yet they face significant hurdles including immune responses to viral or nanoparticle delivery vectors, ethical and safety considerations, and technical complexity in delivery to the injured CNS. In practice, the optimal approach may depend on the context: small molecules might be favored for acute, systemic intervention, whereas EV-based or gene therapies could offer more specific, sustained effects in subacute or chronic phases. Ultimately, a combination of these strategies may be required to achieve optimal neuroprotection and functional recovery after SCI.

Despite these advances, critical knowledge gaps persist. Foremost, the cell-type-specific contribution to the pyroptotic burden is poorly defined: infiltrating macrophages, astrocytes, oligodendrocytes and neurons may die via pyroptosis alongside microglia (2, 133). Most studies focus on inflammasome activation in mixed glial populations or whole spinal tissue, lacking resolution of pyroptosis dynamics in specific cell types (134). This obscures the relative contributions of microglial versus other cell pyroptosis to secondary injury. For instance, conflating microglia with monocyte-derived macrophages in analyses may mask functional differences. Advanced *in vivo* tracing and purified *in vitro* models are needed to dissect cell-specific mechanisms.

Second, functional distinctions among Gasdermin (GSDM) family members in SCI remain poorly understood (67). While GSDMD is widely recognized as the executor of inflammasome-mediated pyroptosis, recent studies suggest GSDME and other family members may mediate pyroptosis via alternative pathways (e.g., caspase-3 activation) (68, 135). In SCI, GSDMD-driven microglial pyroptosis is well-documented, but evidence for roles of GSDME, GSDMC, or other “non-canonical” pyroptosis pathways in neuronal or glial death is lacking. This gap limits our holistic understanding of pyroptosis networks in SCI.

Third, the optimal therapeutic window for pyroptosis inhibition requires clarification (136). Secondary injury spans acute, subacute, and chronic phases, with pyroptosis activity and tissue impacts likely varying across stages (137, 138). While inflammasome components and cleaved GSDMD surge in early injury (hours to days), long-term pyroptosis activity (weeks to chronic phases) remains inconsistently reported (139). Endogenous regulatory mechanisms may partially suppress pyroptosis but fail to halt progressive damage (140, 141). Timing interventions is thus critical: early blockade might disrupt essential immune clearance, whereas delayed action risks irreversible inflammatory cascades. Systematic temporal mapping of pyroptosis activity and intervention efficacy is needed to define optimal clinical windows.

In summary, converging advances in multi-omic analytics, bio-engineered delivery systems and genome editing are poised to transform our mechanistic understanding of microglial pyroptosis into clinically actionable therapies, with the potential to lessen the lifelong disability burden of SCI.

## References

[1] Cowan H Lakra C Desai M. Autonomic dysreflexia in spinal cord injury. Bmj. (2020) 371:m3596. doi: 10.1136/bmj.m3596, PMID: 33008797

[2] Li C Wu Z Zhou L Shao J Hu x Xu W. Temporal and spatial cellular and molecular pathological alterations with single-cell resolution in the adult spinal cord after injury. Signal Transduct Target Ther. (2022) 7:65. doi: 10.1038/s41392-022-00885-4, PMID: 35232960 PMC8888618

[3] Ryan F Blex C Ngo TD Kopp MA Michalke B Venkataramani V. Ferroptosis inhibitor improves outcome after early and delayed treatment in mild spinal cord injury. Acta Neuropathol. (2024) 147:106. doi: 10.1007/s00401-024-02758-2, PMID: 38907771 PMC11193702

[4] Guha L Kumar H. Drug repurposing for spinal cord injury: progress towards therapeutic intervention for primary factors and secondary complications. Pharmaceut Med. (2023) 37:463–90. doi: 10.1007/s40290-023-00499-3, PMID: 37698762

[5] Zavvarian MM Modi AD Sadat S Hong J Fehlings MG. Translational relevance of secondary intracellular signaling cascades following traumatic spinal cord injury. Int J Mol Sci. (2024) 25:5708. doi: 10.3390/ijms25115708, PMID: 38891894 PMC11172219

[6] Liu FS Huang HL Deng LX Zhang QS Wang XB Li J. Identification and bioinformatics analysis of genes associated with pyroptosis in spinal cord injury of rat and mouse. Sci Rep. (2024) 14:14023. doi: 10.1038/s41598-024-64843-6, PMID: 38890348 PMC11189416

[7] Li X Fu J Guan M Shi H Pan W Lou X. Biochanin A attenuates spinal cord injury in rats during early stages by inhibiting oxidative stress and inflammasome activation. Neural Regener Res. (2024) 19:2050–6. doi: 10.4103/1673-5374.390953, PMID: 38227535 PMC11040286

[8] Zhou H Qian Q Chen Q Chen T Wu C Chen L. Enhanced mitochondrial targeting and inhibition of pyroptosis with multifunctional metallopolyphenol nanoparticles in intervertebral disc degeneration. Small. (2024) 20:e2308167. doi: 10.1002/smll.202308167, PMID: 37953455

[9] Fu SP Chen SY Pang QM Zhang M Wu XC Wan X. Advances in the research of the role of macrophage/microglia polarization-mediated inflammatory response in spinal cord injury. Front Immunol. (2022) 13:1014013. doi: 10.3389/fimmu.2022.1014013, PMID: 36532022 PMC9751019

[10] Ramos D Cruz CD. Involvement of microglia in chronic neuropathic pain associated with spinal cord injury - a systematic review. Rev Neurosci. (2023) 34:933–50. doi: 10.1515/revneuro-2023-0031, PMID: 37490300

[11] Zhang C Kang J Zhang X Zhang Y Huang N Ning B. Spatiotemporal dynamics of the cellular components involved in glial scar formation following spinal cord injury. BioMed Pharmacother. (2022) 153:113500. doi: 10.1016/j.biopha.2022.113500, PMID: 36076590

[12] Al Mamun A Wu Y Monalisa I Jia C Zhou K Munir F. Role of pyroptosis in spinal cord injury and its therapeutic implications. J Adv Res. (2021) 28:97–109. doi: 10.1016/j.jare.2020.08.004, PMID: 33364048 PMC7753222

[13] Oladapo A Jackson T Menolascino J Periyasamy P. Role of pyroptosis in the pathogenesis of various neurological diseases. Brain Behav Immun. (2024) 117:428–46. doi: 10.1016/j.bbi.2024.02.001, PMID: 38336022 PMC10911058

[14] Hu X Xu W Ren Y Wang Z He X Huang R. Spinal cord injury: molecular mechanisms and therapeutic interventions. Signal Transduct Target Ther. (2023) 8:245. doi: 10.1038/s41392-023-01477-6, PMID: 37357239 PMC10291001

[15] Cantó-Santos J Grau-Junyent JM Garrabou G. The impact of mitochondrial deficiencies in neuromuscular diseases. Antioxidants (Basel). (2020) 9:964. doi: 10.3390/antiox9100964, PMID: 33050147 PMC7600520

[16] Zheng B Tuszynski MH. Regulation of axonal regeneration after mammalian spinal cord injury. Nat Rev Mol Cell Biol. (2023) 24:396–413. doi: 10.1038/s41580-022-00562-y, PMID: 36604586

[17] Shi Z Yuan S Shi L Li j Ning B Kong X. Programmed cell death in spinal cord injury pathogenesis and therapy. Cell Prolif. (2021) 54:e12992. doi: 10.1111/cpr.12992, PMID: 33506613 PMC7941236

[18] Borst K Dumas AA Prinz M. Microglia: Immune and non-immune functions. Immunity. (2021) 54:2194–208. doi: 10.1016/j.immuni.2021.09.014, PMID: 34644556

[19] Vidal-Itriago A Radford RAW Aramideh JA Maurel C Scherer NM Don EK. Microglia morphophysiological diversity and its implications for the CNS. Front Immunol. (2022) 13:997786. doi: 10.3389/fimmu.2022.997786, PMID: 36341385 PMC9627549

[20] Hasel P Aisenberg WH Bennett FC Liddelow SA. Molecular and metabolic heterogeneity of astrocytes and microglia. Cell Metab. (2023) 35:555–70. doi: 10.1016/j.cmet.2023.03.006, PMID: 36958329

[21] Devanney NA Stewart AN Gensel JC. Microglia and macrophage metabolism in CNS injury and disease: The role of immunometabolism in neurodegeneration and neurotrauma. Exp Neurol. (2020) 329:113310. doi: 10.1016/j.expneurol.2020.113310, PMID: 32289316 PMC7237336

[22] Long Y Li XQ Deng J Ye Q Li D Ma Y. Modulating the polarization phenotype of microglia - A valuable strategy for central nervous system diseases. Ageing Res Rev. (2024) 93:102160. doi: 10.1016/j.arr.2023.102160, PMID: 38065225

[23] Javanmehr N Saleki K Alijanizadeh P Rezaei N. Microglia dynamics in aging-related neurobehavioral and neuroinflammatory diseases. J Neuroinflamm. (2022) 19:273. doi: 10.1186/s12974-022-02637-1, PMID: 36397116 PMC9669544

[24] Li Q Zhao Y Guo H Li Q Yan C Li Y. Impaired lipophagy induced-microglial lipid droplets accumulation contributes to the buildup of TREM1 in diabetes-associated cognitive impairment. Autophagy. (2023) 19:2639–56. doi: 10.1080/15548627.2023.2213984, PMID: 37204119 PMC10472854

[25] Choi BR Johnson KR Maric D McGavern DB. Monocyte-derived IL-6 programs microglia to rebuild damaged brain vasculature. Nat Immunol. (2023) 24:1110–23. doi: 10.1038/s41590-023-01521-1, PMID: 37248420 PMC11531796

[26] Xia DY Yuan JL Jiang XC Qi M Lai NS Wu LY. SIRT1 promotes M2 microglia polarization via reducing ROS-mediated NLRP3 inflammasome signaling after subarachnoid hemorrhage. Front Immunol. (2021) 12:770744. doi: 10.3389/fimmu.2021.770744, PMID: 34899720 PMC8653696

[27] Zhang L Wei W Ai X Kilic E Hermann DM Venkataramani V. Extracellular vesicles from hypoxia-preconditioned microglia promote angiogenesis and repress apoptosis in stroke mice via the TGF-β/Smad2/3 pathway. Cell Death Dis. (2021) 12:1068. doi: 10.1038/s41419-021-04363-7, PMID: 34753919 PMC8578653

[28] Shemer A Scheyltjens I Frumer GR Kim JS Grozovski J Ayanaw S. Interleukin-10 prevents pathological microglia hyperactivation following peripheral endotoxin challenge. Immunity. (2020) 53:1033–49.e7. doi: 10.1016/j.immuni.2020.09.018, PMID: 33049219

[29] Guedes JR Ferreira PA Costa J Laranjo M Pinto MJ Reis T. IL-4 shapes microglia-dependent pruning of the cerebellum during postnatal development. Neuron. (2023) 111:3435–49.e8. doi: 10.1016/j.neuron.2023.09.031, PMID: 37918358

[30] Paolicelli RC Sierra A Stevens B Tremblay ME Aguzzi A Ajami B. Microglia states and nomenclature: A field at its crossroads. Neuron. (2022) 110:3458–83. doi: 10.1016/j.neuron.2022.10.020, PMID: 36327895 PMC9999291

[31] Peng R Zhang L Xie Y Guo S Cao X Yang M. Spatial multi-omics analysis of the microenvironment in traumatic spinal cord injury: a narrative review. Front Immunol. (2024) 15:1432841. doi: 10.3389/fimmu.2024.1432841, PMID: 39267742 PMC11390538

[32] Wang J Xu L Lin W Yao Y Li H Shen G. Single-cell transcriptome analysis reveals the immune heterogeneity and the repopulation of microglia by Hif1α in mice after spinal cord injury. Cell Death Dis. (2022) 13:432. doi: 10.1038/s41419-022-04864-z, PMID: 35504882 PMC9065023

[33] Kracht L Borggrewe M Eskandar S Brouwer N Chuva de Sousa Lopes SM Laman JD. Human fetal microglia acquire homeostatic immune-sensing properties early in development. Science. (2020) 369:530–7. doi: 10.1126/science.aba5906, PMID: 32732419

[34] Skinnider MA Rogalski J Tigchelaar S Manouchehri N Prudova A Jackson AM. Proteomic portraits reveal evolutionarily conserved and divergent responses to spinal cord injury. Mol Cell Proteomics. (2021) 20:100096. doi: 10.1016/j.mcpro.2021.100096, PMID: 34129941 PMC8260874

[35] Saas P Vetter M Maraux M Bonnefoy F Perruche S. Resolution therapy: Harnessing efferocytic macrophages to trigger the resolution of inflammation. Front Immunol. (2022) 13:1021413. doi: 10.3389/fimmu.2022.1021413, PMID: 36389733 PMC9651061

[36] Gerlach BD Ampomah PB Yurdagul A,JR Liu C Lauring MC Wang X. Efferocytosis induces macrophage proliferation to help resolve tissue injury. Cell Metab. (2021) 33:2445–63.e8. doi: 10.1016/j.cmet.2021.10.015, PMID: 34784501 PMC8665147

[37] Soliman E Leonard J Basso EKG Gershenson I Ju J Mills J. Efferocytosis is restricted by axon guidance molecule EphA4 via ERK/Stat6/MERTK signaling following brain injury. J Neuroinflamm. (2023) 20:256. doi: 10.1186/s12974-023-02940-5, PMID: 37941008 PMC10633953

[38] Nguyen LT Aprico A Nwoke E Walsh AD Blades F Avneri R. Mertk-expressing microglia influence oligodendrogenesis and myelin modelling in the CNS. J Neuroinflamm. (2023) 20:253. doi: 10.1186/s12974-023-02921-8, PMID: 37926818 PMC10626688

[39] Chen J Zeng X Wang L Zhang W Li G Cheng X. Mutual regulation of microglia and astrocytes after Gas6 inhibits spinal cord injury. Neural Regener Res. (2025) 20:557–73. doi: 10.4103/nrr.Nrr-d-23-01130, PMID: 38819067 PMC11317951

[40] Cai W Dai X Chen J Zhang W Li G Cheng X. STAT6/Arg1 promotes microglia/macrophage efferocytosis and inflammation resolution in stroke mice. JCI Insight. (2019) 4. doi: 10.1172/jci.insight.131355, PMID: 31619589 PMC6824303

[41] Zhang G Li Q Tao W Qin P Chen J Yang H. Sigma-1 receptor-regulated efferocytosis by infiltrating circulating macrophages/microglial cells protects against neuronal impairments and promotes functional recovery in cerebral ischemic stroke. Theranostics. (2023) 13:543–59. doi: 10.7150/thno.77088, PMID: 36632219 PMC9830433

[42] Chen Y Kou Y Ni Y Yang H Xu C Fan H. Microglia efferocytosis: an emerging mechanism for the resolution of neuroinflammation in Alzheimer's disease. J Neuroinflamm. (2025) 22:96. doi: 10.1186/s12974-025-03428-0, PMID: 40159486 PMC11955113

[43] Liu X Wang J Jin J Hu Q Zhao T Wang J. S100A9 deletion in microglia/macrophages ameliorates brain injury through the STAT6/PPARγ pathway in ischemic stroke. CNS Neurosci Ther. (2024) 30:e14881. doi: 10.1111/cns.14881, PMID: 39107960 PMC11303267

[44] Wu J Tang J Zhang L Wang W Li Z Zhou L. Biomimetic "Trojan horse" Fibers modulate innate immunity cascades for nerve regeneration. ACS Nano. (2025) 19:781–802. doi: 10.1021/acsnano.4c12036, PMID: 39708371 PMC11752508

[45] Gu HY Liu N. Mechanism of effect and therapeutic potential of NLRP3 inflammasome in spinal cord injury. Exp Neurol. (2025) 384:115059. doi: 10.1016/j.expneurol.2024.115059, PMID: 39571746

[46] Wang H Zhang Y Ma X Wang W Xu X Huang M. Spinal TLR4/P2X7 receptor-dependent NLRP3 inflammasome activation contributes to the development of tolerance to morphine-induced antinociception. J Inflammation Res. (2020) 13:571–82. doi: 10.2147/jir.S266995, PMID: 33061523 PMC7522404

[47] Mo Y Chen K. Review: The role of HMGB1 in spinal cord injury. Front Immunol. (2022) 13:1094925. doi: 10.3389/fimmu.2022.1094925, PMID: 36713448 PMC9877301

[48] Nakahira K Haspel JA Rathinam VA Lee SJ Dolinay T Lam HC. Autophagy proteins regulate innate immune responses by inhibiting the release of mitochondrial DNA mediated by the NALP3 inflammasome. Nat Immunol. (2011) 12:222–30. doi: 10.1038/ni.1980, PMID: 21151103 PMC3079381

[49] Lin QC Wang J Wang XL Pan C Jin SW Char S. Hippocampal HDAC6 promotes POCD by regulating NLRP3-induced microglia pyroptosis via HSP90/HSP70 in aged mice. Biochim Biophys Acta Mol Basis Dis. (2024) 1870:167137. doi: 10.1016/j.bbadis.2024.167137, PMID: 38527593

[50] Qu J Tao XY Teng P Zhang Y Guo CL Hu L. Blocking ATP-sensitive potassium channel alleviates morphine tolerance by inhibiting HSP70-TLR4-NLRP3-mediated neuroinflammation. J Neuroinflamm. (2017) 14:228. doi: 10.1186/s12974-017-0997-0, PMID: 29178967 PMC5702153

[51] Ha JS Choi HR Kim IS Kim EA Cho SW Yang SJ. Hypoxia-induced S100A8 expression activates microglial inflammation and promotes neuronal apoptosis. Int J Mol Sci. (2021) 22. doi: 10.3390/ijms22031205, PMID: 33530496 PMC7866104

[52] Thapa A Adamiak M Bujko K Ratajczak J Abdel-Latif AK Kucia M. Danger-associated molecular pattern molecules take unexpectedly a central stage in Nlrp3 inflammasome-caspase-1-mediated trafficking of hematopoietic stem/progenitor cells. Leukemia. (2021) 35:2658–71. doi: 10.1038/s41375-021-01158-9, PMID: 33623143 PMC8410600

[53] Kodi T Sankhe R Gopinathan A Nandakumar K Kishore A. New insights on NLRP3 inflammasome: mechanisms of activation, inhibition, and epigenetic regulation. J Neuroimmune Pharmacol. (2024) 19:7. doi: 10.1007/s11481-024-10101-5, PMID: 38421496 PMC10904444

[54] He Y Hara H Núñez G. Mechanism and regulation of NLRP3 inflammasome activation. Trends Biochem Sci. (2016) 41:1012–21. doi: 10.1016/j.tibs.2016.09.002, PMID: 27669650 PMC5123939

[55] Chen J Shen Y Shao X Wu W. An emerging role of inflammasomes in spinal cord injury and spinal cord tumor. Front Immunol. (2023) 14:1119591. doi: 10.3389/fimmu.2023.1119591, PMID: 36969234 PMC10033975

[56] Tang T Lang X Xu C Wang X Gong T Yang Y. CLICs-dependent chloride efflux is an essential and proximal upstream event for NLRP3 inflammasome activation. Nat Commun. (2017) 8:202. doi: 10.1038/s41467-017-00227-x, PMID: 28779175 PMC5544706

[57] Xiao X Chen XY Dong YH Dong HR Zhou LN Ding YQ. Pre-treatment of rapamycin transformed M2 microglia alleviates traumatic cervical spinal cord injury via AIM2 signaling pathway *in vitro* and *in vivo* . Int Immunopharmacol. (2023) 121:110394. doi: 10.1016/j.intimp.2023.110394, PMID: 37295027

[58] Zuo X Ju C Zhang Z Wei X Ma Y Song Z. Photobiomodulation regulates inflammation and autophagy in spinal cord injury through NLRP3/Caspase-1/IL-1β pathway by targeting TLR2. Mol Immunol. (2025) 182:1–10. doi: 10.1016/j.molimm.2025.03.014, PMID: 40157277

[59] Shi J Zhao Y Wang Y Gao W Ding J Li P. Inflammatory caspases are innate immune receptors for intracellular LPS. Nature. (2014) 514:187–92. doi: 10.1038/nature13683, PMID: 25119034

[60] Shi X Sun Q Hou Y Zeng H Cao Y Dong M. Recognition and maturation of IL-18 by caspase-4 noncanonical inflammasome. Nature. (2023) 624:442–50. doi: 10.1038/s41586-023-06742-w, PMID: 37993714

[61] Kayagaki N Stowe IB Lee BL O'Rourke K Anderson K Warming S. Caspase-11 cleaves gasdermin D for non-canonical inflammasome signalling. Nature. (2015) 526:666–71. doi: 10.1038/nature15541, PMID: 26375259

[62] Zhou Y Chai Z Pandeya A Yang L Zhang Y Zhang G. Caspase-11 and NLRP3 exacerbate systemic Klebsiella infection through reducing mitochondrial ROS production. Front Immunol. (2025) 16:1516120. doi: 10.3389/fimmu.2025.1516120, PMID: 40034692 PMC11873083

[63] Yang D He Y Muñoz-Planillo R Liu Q Núñez G. Caspase-11 requires the pannexin-1 channel and the purinergic P2X7 pore to mediate pyroptosis and endotoxic shock. Immunity. (2015) 43:923–32. doi: 10.1016/j.immuni.2015.10.009, PMID: 26572062 PMC4795157

[64] Li Z Rong Y Zhang Y. MiR-335 improves functional recovery in rats after spinal cord injury and protects PC12 cells against injury via the SPI-bax/caspase-3 axis. Spine (Phila Pa 1976). (2024) 49:583–93. doi: 10.1097/brs.0000000000004862, PMID: 38167229

[65] Zhao W Li H Hou Y Jin Y Zhang L. Combined administration of poly-ADP-ribose polymerase-1 and caspase-3 inhibitors alleviates neuronal apoptosis after spinal cord injury in rats. World Neurosurg. (2019) 127:e346–e52. doi: 10.1016/j.wneu.2019.03.116, PMID: 30904799

[66] Wang Y Gao W Shi X Ding J Liu W He H. Chemotherapy drugs induce pyroptosis through caspase-3 cleavage of a gasdermin. Nature. (2017) 547:99–103. doi: 10.1038/nature22393, PMID: 28459430

[67] Zhu C Xu S Jiang R Yu Y Bian J Zou Z. The gasdermin family: emerging therapeutic targets in diseases. Signal Transduct Target Ther. (2024) 9:87. doi: 10.1038/s41392-024-01801-8, PMID: 38584157 PMC10999458

[68] Wu C Wang L Chen S Shi L Liu M Tu P. Iron induces B cell pyroptosis through Tom20-Bax-caspase-gasdermin E signaling to promote inflammation post-spinal cord injury. J Neuroinflamm. (2023) 20:171. doi: 10.1186/s12974-023-02848-0, PMID: 37480037 PMC10362643

[69] Mckenzie BA Fernandes JP Doan MAL Schmitt LM Branton WG Power C. Activation of the executioner caspases-3 and -7 promotes microglial pyroptosis in models of multiple sclerosis. J Neuroinflamm. (2020) 17:253. doi: 10.1186/s12974-020-01902-5, PMID: 32861242 PMC7456507

[70] Hou J Zhao R Xia W Chang CW You Y Hsu JM. PD-L1-mediated gasdermin C expression switches apoptosis to pyroptosis in cancer cells and facilitates tumour necrosis. Nat Cell Biol. (2020) 22:1264–75. doi: 10.1038/s41556-020-0575-z, PMID: 32929201 PMC7653546

[71] Zhang JY Zhou B Sun RY Ai YL Cheng K Li FN. The metabolite α-KG induces GSDMC-dependent pyroptosis through death receptor 6-activated caspase-8. Cell Res. (2021) 31:980–97. doi: 10.1038/s41422-021-00506-9, PMID: 34012073 PMC8410789

[72] Chen KW Demarco B Heilig R Shkarina K Boettcher A Farady CJ. Extrinsic and intrinsic apoptosis activate pannexin-1 to drive NLRP3 inflammasome assembly. EMBO J. (2019) 38. doi: 10.15252/embj.2019101638, PMID: 30902848 PMC6517827

[73] Muendlein HI Connolly WM Leiriao J Nolan MA Judge J Smirnova I. TNF switches homeostatic efferocytosis to lytic caspase-8-dependent pyroptosis and IL-1β maturation. Sci Immunol. (2025) 10:eadq0043. doi: 10.1126/sciimmunol.adq0043, PMID: 40540586

[74] Zhou H Li Z Jing S Wang B Ye Z Xiong W. Repair spinal cord injury with a versatile anti-oxidant and neural regenerative nanoplatform. J Nanobiotechnology. (2024) 22:351. doi: 10.1186/s12951-024-02610-5, PMID: 38902789 PMC11188197

[75] Liu Z Shi J Tu K Ma H Chen J Xiang X. GPx3 promotes functional recovery after spinal cord injury by inhibiting microglial pyroptosis through IRAK4/ROS/NLRP3 axis. Antioxid Redox Signal. (2025) 42:711–29. doi: 10.1089/ars.2024.0618, PMID: 39895340

[76] Ren Z Liang W Sheng J Xun C Xu T Cao R. Gal-3 is a potential biomarker for spinal cord injury and Gal-3 deficiency attenuates neuroinflammation through ROS/TXNIP/NLRP3 signaling pathway. Biosci Rep. (2019) 39. doi: 10.1042/bsr20192368, PMID: 31763668 PMC6923351

[77] Liu Z Tu K Zou P Liao C Ding R Huang Z. Hesperetin ameliorates spinal cord injury by inhibiting NLRP3 inflammasome activation and pyroptosis through enhancing Nrf2 signaling. Int Immunopharmacol. (2023) 118:110103. doi: 10.1016/j.intimp.2023.110103, PMID: 37001385

[78] Yang S Guan Y Zheng C Xia X Ma X Jiang J. FOXO3-induced microRNA-128-3p promotes the progression of spinal cord injury in mice via regulating NLRP3 inflammasome-mediated pyroptosis. Front Immunol. (2025) 16:1526721. doi: 10.3389/fimmu.2025.1526721, PMID: 40061945 PMC11885150

[79] Zhang B Yu J Bao L Feng D Qin Y Fan D. Cynarin inhibits microglia-induced pyroptosis and neuroinflammation via Nrf2/ROS/NLRP3 axis after spinal cord injury. Inflammation Res. (2024) 73:1981–94. doi: 10.1007/s00011-024-01945-x, PMID: 39340662

[80] Jiang W He F Ding G Wu J. Elamipretide reduces pyroptosis and improves functional recovery after spinal cord injury. CNS Neurosci Ther. (2023) 29:2843–56. doi: 10.1111/cns.14221, PMID: 37081763 PMC10493668

[81] Zhou LY Yao M Tian ZR Liu SF Song YJ Ye J. Muscone suppresses inflammatory responses and neuronal damage in a rat model of cervical spondylotic myelopathy by regulating Drp1-dependent mitochondrial fission. J Neurochem. (2020) 155:154–76. doi: 10.1111/jnc.15011, PMID: 32215908

[82] Bai M Cui Y Sang Z Gao S Zhao H Mei X. Zinc ions regulate mitochondrial quality control in neurons under oxidative stress and reduce PANoptosis in spinal cord injury models via the Lgals3-Bax pathway. Free Radic Biol Med. (2024) 221:169–80. doi: 10.1016/j.freeradbiomed.2024.05.037, PMID: 38782079

[83] Rabchevsky AG Michael FM Patel SP. Mitochondria focused neurotherapeutics for spinal cord injury. Exp Neurol. (2020) 330:113332. doi: 10.1016/j.expneurol.2020.113332, PMID: 32353464 PMC9164988

[84] Slater PG Domínguez-Romero ME Villarreal M Eisner V Larraín J. Mitochondrial function in spinal cord injury and regeneration. Cell Mol Life Sci. (2022) 79:239. doi: 10.1007/s00018-022-04261-x, PMID: 35416520 PMC11072423

[85] Wu C Chen H Zhuang R Zhang H Wang Y Hu X. Betulinic acid inhibits pyroptosis in spinal cord injury by augmenting autophagy via the AMPK-mTOR-TFEB signaling pathway. Int J Biol Sci. (2021) 17:1138–52. doi: 10.7150/ijbs.57825, PMID: 33867836 PMC8040310

[86] Chen K Ying J Zhu J Chen L Liu R Jing M. Urolithin A alleviates NLRP3 inflammasome activation and pyroptosis by promoting microglial mitophagy following spinal cord injury. Int Immunopharmacol. (2025) 148:114057. doi: 10.1016/j.intimp.2025.114057, PMID: 39827665

[87] Ran N Li W Zhang R Lin C Zhang J Wei Z. Autologous exosome facilitates load and target delivery of bioactive peptides to repair spinal cord injury. Bioact Mater. (2023) 25:766–82. doi: 10.1016/j.bioactmat.2022.07.002, PMID: 37056263 PMC10086682

[88] Welsh JA Goberdhan DCI O'driscoll L Buzas EI Blenkiron C Bussolati B. Minimal information for studies of extracellular vesicles (MISEV2023): From basic to advanced approaches. J Extracell Vesicles. (2024) 13:e12404. doi: 10.1002/jev2.12404, PMID: 38326288 PMC10850029

[89] Liu WZ Ma ZJ Li JR Kang XW. Mesenchymal stem cell-derived exosomes: therapeutic opportunities and challenges for spinal cord injury. Stem Cell Res Ther. (2021) 12:102. doi: 10.1186/s13287-021-02153-8, PMID: 33536064 PMC7860030

[90] Debbi L Guo S Safina D Levenberg S. Boosting extracellular vesicle secretion. Biotechnol Adv. (2022) 59:107983. doi: 10.1016/j.bioteChadv.2022.107983, PMID: 35588952 PMC9420194

[91] Xie Y Sun Y Liu Y Zhao J Liu Q Xu J. Targeted delivery of RGD-CD146(+)CD271(+) human umbilical cord mesenchymal stem cell-derived exosomes promotes blood-spinal cord barrier repair after spinal cord injury. ACS Nano. (2023) 17:18008–24. doi: 10.1021/acsnano.3c04423, PMID: 37695238

[92] Xiong W Li C Kong G Zeng Q Wang S Yin G. Treg cell-derived exosomes miR-709 attenuates microglia pyroptosis and promotes motor function recovery after spinal cord injury. J Nanobiotechnology. (2022) 20:529. doi: 10.1186/s12951-022-01724-y, PMID: 36514078 PMC9745961

[93] Gu J Wu J Wang C Xu Z Jin Z Yan D. BMSCs-derived exosomes inhibit macrophage/microglia pyroptosis by increasing autophagy through the miR-21a-5p/PELI1 axis in spinal cord injury. Aging (Albany NY). (2024) 16:5184–206. doi: 10.18632/aging.205638, PMID: 38466640 PMC11006467

[94] Liu J Kong G Lu C Wang J Li W Lv Z. IPSC-NSCs-derived exosomal let-7b-5p improves motor function after spinal cord Injury by modulating microglial/macrophage pyroptosis. J Nanobiotechnology. (2024) 22:403. doi: 10.1186/s12951-024-02697-w, PMID: 38982427 PMC11232148

[95] Xu S Wang J Jiang J Song J Zhu W Zhang F. TLR4 promotes microglial pyroptosis via lncRNA-F630028O10Rik by activating PI3K/AKT pathway after spinal cord injury. Cell Death Dis. (2020) 11:693. doi: 10.1038/s41419-020-02824-z, PMID: 32826878 PMC7443136

[96] Liu T Ma Z Liu L Pei Y Wu Q Xu S. Conditioned medium from human dental pulp stem cells treats spinal cord injury by inhibiting microglial pyroptosis. Neural Regener Res. (2024) 19:1105–11. doi: 10.4103/1673-5374.385309, PMID: 37862215 PMC10749599

[97] Zhang Y Zhang W Liu T Ma Z Zhang W Guan Y. Upregulation of circ0000381 attenuates microglial/macrophage pyroptosis after spinal cord injury. Neural Regener Res. (2024) 19:1360–6. doi: 10.4103/1673-5374.386399, PMID: 37905886 PMC11467933

[98] Sheng Y Zhou X Wang J Shen H Wu S Guo W. MSC derived EV loaded with miRNA-22 inhibits the inflammatory response and nerve function recovery after spinal cord injury in rats. J Cell Mol Med. (2021) 25:10268–78. doi: 10.1111/jcmm.16965, PMID: 34609045 PMC8572783

[99] Wang C Zhang J Chen W Gao L He J Xia Y. Exosomal lncRNA RMRP-shuttled by Olfactory Mucosa-Mesenchymal Stem Cells Suppresses Microglial Pyroptosis to Improve Spinal Cord Injury via EIF4A3/SIRT1. Mol Neurobiol. (2025) 1–16. doi: 10.1007/s12035-025-04756-1, PMID: 39982689

[100] Zhang D Mao F Wang S Wu H Wang S Liao Y. Role of transcription factor nrf2 in pyroptosis in spinal cord injury by regulating GSDMD. Neurochem Res. (2023) 48:172–87. doi: 10.1007/s11064-022-03719-5, PMID: 36040608

[101] Liu Z Yao X Jiang W Li W Zhu S Liao C. Advanced oxidation protein products induce microglia-mediated neuroinflammation via MAPKs-NF-κB signaling pathway and pyroptosis after secondary spinal cord injury. J Neuroinflamm. (2020) 17:90. doi: 10.1186/s12974-020-01751-2, PMID: 32192500 PMC7082940

[102] Jiang Z Zeng Z He H Li M Lan Y Hui J. Lycium barbarum glycopeptide alleviates neuroinflammation in spinal cord injury via modulating docosahexaenoic acid to inhibiting MAPKs/NF-kB and pyroptosis pathways. J Transl Med. (2023) 21:770. doi: 10.1186/s12967-023-04648-9, PMID: 37907930 PMC10617163

[103] Li F Sun X Sun K Kong F Jiang X Kong Q. Lupenone improves motor dysfunction in spinal cord injury mice through inhibiting the inflammasome activation and pyroptosis in microglia via the nuclear factor kappa B pathway. Neural Regener Res. (2024) 19:1802–11. doi: 10.4103/1673-5374.389302, PMID: 38103247 PMC10960275

[104] Li D Dai Y Li Z Bi H Li H Wang Y. Resveratrol upregulates miR-124-3p expression to target DAPK1, regulating the NLRP3/caspase-1/GSDMD pathway to inhibit pyroptosis and alleviate spinal cord injury. J Cell Mol Med. (2025) 29:e70338. doi: 10.1111/jcmm.70338, PMID: 39833100 PMC11745821

[105] Dai W Wang X Teng H Li C Wang B Wang J. Celastrol inhibits microglial pyroptosis and attenuates inflammatory reaction in acute spinal cord injury rats. Int Immunopharmacol. (2019) 66:215–23. doi: 10.1016/j.intimp.2018.11.029, PMID: 30472522

[106] Liu Z Yao X Sun B Jiang W Liao C Dai X. Pretreatment with kaempferol attenuates microglia-mediate neuroinflammation by inhibiting MAPKs-NF-κB signaling pathway and pyroptosis after secondary spinal cord injury. Free Radic Biol Med. (2021) 168:142–54. doi: 10.1016/j.freeradbiomed.2021.03.037, PMID: 33823244

[107] Yan R Yuan Y Shi C Li Y Li Y Wang W. Kanglexin attenuates spinal cord injury by modulating pyroptosis and polarization via the PKA/NF-κB signaling pathway. Int Immunopharmacol. (2025) 153:114401. doi: 10.1016/j.intimp.2025.114401, PMID: 40101425

[108] Hu Z Xuan L Wu T Jiang N Liu X Chang J. Taxifolin attenuates neuroinflammation and microglial pyroptosis via the PI3K/Akt signaling pathway after spinal cord injury. Int Immunopharmacol. (2023) 114:109616. doi: 10.1016/j.intimp.2022.109616, PMID: 36700780

[109] Wang B Chang M Zhang R Wo J Wu B Zhang H. Spinal cord injury target-immunotherapy with TNF-α autoregulated and feedback-controlled human umbilical cord mesenchymal stem cell derived exosomes remodelled by CRISPR/Cas9 plasmid. Biomater Adv. (2022) 133:112624. doi: 10.1016/j.msec.2021.112624, PMID: 35525736

[110] Chen Q Chuai G Zhang H Tang J Duan L Guan H. Genome-wide CRISPR off-target prediction and optimization using RNA-DNA interaction fingerprints. Nat Commun. (2023) 14:7521. doi: 10.1038/s41467-023-42695-4, PMID: 37980345 PMC10657421

[111] Xu S Wang J Zhong J Shao M Jiang J Song J. CD73 alleviates GSDMD-mediated microglia pyroptosis in spinal cord injury through PI3K/AKT/Foxo1 signaling. Clin Transl Med. (2021) 11:e269. doi: 10.1002/ctm2.269, PMID: 33463071 PMC7774461

[112] Wang J Zhang F Xu H Yang H Shao M Xu S. TLR4 aggravates microglial pyroptosis by promoting DDX3X-mediated NLRP3 inflammasome activation via JAK2/STAT1 pathway after spinal cord injury. Clin Transl Med. (2022) 12:e894. doi: 10.1002/ctm2.894, PMID: 35692100 PMC9189419

[113] He X Deng B Zhang C Zhang G Yang F Zhu D. HSPA1A inhibits pyroptosis and neuroinflammation after spinal cord injury via DUSP1 inhibition of the MAPK signaling pathway. Mol Med. (2025) 31:53. doi: 10.1186/s10020-025-01086-9, PMID: 39924492 PMC11809008

[114] Li D Liu S Lu X Gong Z Wang H Xia X. The circadian clock gene bmal1 regulates microglial pyroptosis after spinal cord injury via NF-κB/MMP9. CNS Neurosci Ther. (2024) 30:e70130. doi: 10.1111/cns.70130, PMID: 39648661 PMC11625957

[115] Xia M Li X Ye S Zhang Q Zhao T Li R. FANCC deficiency mediates microglial pyroptosis and secondary neuronal apoptosis in spinal cord contusion. Cell Biosci. (2022) 12:82. doi: 10.1186/s13578-022-00816-4, PMID: 35659106 PMC9164466

[116] Zhang W Lu Y Shen R Wu Y Liu C Fang X. Inhibiting ceramide synthase 5 expression in microglia decreases neuroinflammation after spinal cord injury. Neural Regener Res. (2025) 20:2955–68. doi: 10.4103/nrr.Nrr-d-23-01933, PMID: 39610106 PMC11826471

[117] Li J Yang Y Zhao C Zhao J Wang X Ye S. Microglial C/EBPβ-Fcgr1 regulatory axis blocking inhibits microglial pyroptosis and improves neurological recovery. J Neuroinflamm. (2025) 22:29. doi: 10.1186/s12974-025-03362-1, PMID: 39891259 PMC11786472

[118] Yang Z Sheng M Wang M Cheng L Sun X. PKR inhibitor protects spinal cord injury through mitigating endoplasmic reticulum stress and pyroptosis. Neurochem Int. (2024) 172:105632. doi: 10.1016/j.neuint.2023.105632, PMID: 37866691

[119] Yu J Feng D Bao L Zhang B. TRIM32 inhibits NEK7 ubiquitylation-dependent microglia pyroptosis after spinal cord injury. Mol Biotechnol. (2025) 67:138–48. doi: 10.1007/s12033-023-00989-4, PMID: 38030945

[120] Zeng ZJ Lin X Yang L Li Y Gao W. Activation of inflammasomes and relevant modulators for the treatment of microglia-mediated neuroinflammation in ischemic stroke. Mol Neurobiol. (2024) 61:10792–804. doi: 10.1007/s12035-024-04225-1, PMID: 38789893

[121] Wu X Wan T Gao X Fu M Duan Y Shen X. Microglia pyroptosis: A candidate target for neurological diseases treatment. Front Neurosci. (2022) 16:922331. doi: 10.3389/fnins.2022.922331, PMID: 35937897 PMC9354884

[122] Wang T Wang L Zhang L Long Y Zhang Y Hou Z. Single-cell RNA sequencing in orthopedic research. Bone Res. (2023) 11:10. doi: 10.1038/s41413-023-00245-0, PMID: 36828839 PMC9958119

[123] Ning B Gao L Liu RH Liu Y Zhang NS Chen ZY. microRNAs in spinal cord injury: potential roles and therapeutic implications. Int J Biol Sci. (2014) 10:997–1006. doi: 10.7150/ijbs.9058, PMID: 25210498 PMC4159691

[124] Silvestro S Mazzon E. MiRNAs as promising translational strategies for neuronal repair and regeneration in spinal cord injury. Cells. (2022) 11:2177. doi: 10.3390/cells11142177, PMID: 35883621 PMC9318426

[125] Lu Y Yang J Wang X Ma Z Li S Liu Z. Research progress in use of traditional Chinese medicine for treatment of spinal cord injury. BioMed Pharmacother. (2020) 127:110136. doi: 10.1016/j.biopha.2020.110136, PMID: 32335299

[126] Wu X Yan Y Zhang Q. Neuroinflammation and modulation role of natural products after spinal cord injury. J Inflammation Res. (2021) 14:5713–37. doi: 10.2147/jir.S329864, PMID: 34764668 PMC8576359

[127] Yang R Pan J Wang Y Xia P Tai M Jiang Z. Application and prospects of somatic cell reprogramming technology for spinal cord injury treatment. Front Cell Neurosci. (2022) 16:1005399. doi: 10.3389/fncel.2022.1005399, PMID: 36467604 PMC9712200

[128] Zeng CW. Stem cell-based approaches for spinal cord injury: the promise of iPSCs. Biol (Basel). (2025) 14:314. doi: 10.3390/biology14030314, PMID: 40136570 PMC11940451

[129] Guo S Perets N Betzer O Ben-Shaul S Sheinin A Michaelevski I. Intranasal delivery of mesenchymal stem cell derived exosomes loaded with phosphatase and tensin homolog siRNA repairs complete spinal cord injury. ACS Nano. (2019) 13:10015–28. doi: 10.1021/acsnano.9b01892, PMID: 31454225

[130] Zeng H Liu N Yang YY Xing HY Liu XX Li F. Lentivirus-mediated downregulation of α-synuclein reduces neuroinflammation and promotes functional recovery in rats with spinal cord injury. J Neuroinflamm. (2019) 16:283. doi: 10.1186/s12974-019-1658-2, PMID: 31888724 PMC6936070

[131] Rong Y Wang J Hu T Shi Z Lang C Liu W. Ginsenoside rg1 regulates immune microenvironment and neurological recovery after spinal cord injury through MYCBP2 delivery via neuronal cell-derived extracellular vesicles. Adv Sci (Weinh). (2024) 11:e2402114. doi: 10.1002/advs.202402114, PMID: 38896802 PMC11336912

[132] Wang S Li G Liang X Wu Z Chen C Zhang F. Small extracellular vesicles derived from altered peptide ligand-loaded dendritic cell act as A therapeutic vaccine for spinal cord injury through eliciting CD4(+) T cell-mediated neuroprotective immunity. Adv Sci (Weinh). (2024) 11:e2304648. doi: 10.1002/advs.202304648, PMID: 38037457 PMC10797491

[133] Li Y He X Kawaguchi R Zhang Y Wang Q Monavarfeshani A. Microglia-organized scar-free spinal cord repair in neonatal mice. Nature. (2020) 587:613–8. doi: 10.1038/s41586-020-2795-6, PMID: 33029008 PMC7704837

[134] Brennan FH Swarts EA Kigerl KA Mifflin KA Guan Z Noble BT. Microglia promote maladaptive plasticity in autonomic circuitry after spinal cord injury in mice. Sci Transl Med. (2024) 16:eadi3259. doi: 10.1126/scitranslmed.adi3259, PMID: 38865485

[135] Neel DV Basu H Gunner G Bergstresser MD Giadone RM Chung H. Gasdermin-E mediates mitochondrial damage in axons and neurodegeneration. Neuron. (2023) 111:1222–40.e9. doi: 10.1016/j.neuron.2023.02.019, PMID: 36917977 PMC10121894

[136] Tansley S Uttam S Ureña Guzmán A Yaqubi M Pacis A Parisien M. Single-cell RNA sequencing reveals time- and sex-specific responses of mouse spinal cord microglia to peripheral nerve injury and links ApoE to chronic pain. Nat Commun. (2022) 13:843. doi: 10.1038/s41467-022-28473-8, PMID: 35149686 PMC8837774

[137] Mattei D Ivanov A Hammer J Ugursu B Schalbetter S Richetto J. Microglia undergo molecular and functional adaptations to dark and light phases in male laboratory mice. Brain Behav Immun. (2024) 120:571–83. doi: 10.1016/j.bbi.2024.07.007, PMID: 38986723

[138] Akhmetzyanova ER Zhuravleva MN Timofeeva AV Tazetdinova LG Garanina EE Rizvanov AA. Severity- and time-dependent activation of microglia in spinal cord injury. Int J Mol Sci. (2023) 24:8294. doi: 10.3390/ijms24098294, PMID: 37176001 PMC10179339

[139] Bai Y Pan Y Liu X. Mechanistic insights into gasdermin-mediated pyroptosis. Nat Rev Mol Cell Biol. (2025) 1–21. doi: 10.1038/s41580-025-00837-0, PMID: 40128620

[140] Wang S Yang L Wu Z Li C Wang S Xiao Z. Ferroptosis-related genes participate in the microglia-induced neuroinflammation of spinal cord injury via NF-κB signaling: evidence from integrated single-cell and spatial transcriptomic analysis. J Transl Med. (2025) 23:43. doi: 10.1186/s12967-025-06095-0, PMID: 39799354 PMC11725224

[141] Zrzavy T Schwaiger C Wimmer I Berger T Bauer J Butovsky O. Acute and non-resolving inflammation associate with oxidative injury after human spinal cord injury. Brain. (2021) 144:144–61. doi: 10.1093/brain/awaa360, PMID: 33578421 PMC7880675

